# Combinatorial inhibition of Plk1 and PKCβ in cancer cells with different p53 status

**DOI:** 10.18632/oncotarget.1897

**Published:** 2014-04-12

**Authors:** Lisa Lange, Sarah Keppner-Witter, Juline Grigat, Birgit Spänkuch

**Affiliations:** ^1^ Friedrich-Schiller-University, CMB, Institute for Biochemistry, Hans-Knöll-Straße 2, 07745 Jena, Germany; ^2^ Eberhard-Karls-University, Department of Gynecology, Calwer Straße 7, 72076 Tübingen, Germany; ^3^ Department of Obstetrics and Gynecology, Medical School, Goethe-University, Theodor-Stern-Kai 7, 60590 Frankfurt am Main, Germany

**Keywords:** polo-like kinase 1, protein-kinase C β, Enzastaurin, SBE13, cell cycle

## Abstract

PKCβ and Plk1 are fascinating targets in cancer therapy. Therefore, we combined Enzastaurin targeting PKCβ and SBE13 targeting Plk1 to test synergistic effects in cells with different p53 status. We analyzed cell proliferation and apoptosis induction, and did Western blot and FACScan analyses to examine the combined PKCβ and Plk1 inhibition. p53-wild-type cells are more resistant to the combinatorial treatment than p53-deficient cells, which displayed a synergistic reduction of cell proliferation after the combination. HeLa, MCF-7 and HCT116^p53wt^ and HCT116^p53-/-^ cells differed in their cell cycle distribution after combinatorial treatment in dependence on a functional p53-dependent G1/S checkpoint (p53-deficient cells showed an enrichment in S and G2/M, p53-wild-type cells in G0/G1 phase). hTERT-RPE1 cells did not show the synergistic effects of cancer cells.

Thus, we demonstrate for the first time that Plk1 inhibition using SBE13 enhances the effects of Enzastaurin in cancer cells. HCT116^p53wt^ and HCT116^p53-/-^ cells confirmed the p53-dependence of different effects after Plk1 and PKCβ inhibition observed in HeLa and MCF-7 cells. Obviously, p53 protects cells from the cytotoxicity of Enzastaurin in combination with SBE13. For that reason this combination can be useful to treat p53-deficient cancers, without displaying toxicity to normal cells, which all have functional p53.

## INTRODUCTION

One of the main research fields in translational cancer research is the search for new therapeutic strategies. Many strategies target one or more cancer-related genes and try to inhibit cancer cell proliferation by inhibiting the expression or activity of these genes. One promising strategy to achieve this goal is the use of small molecule kinase inhibitors, which represent essential tools in basic and translational research.

One cancer related gene family is the Protein Kinase C family (PKC) and one crucial family member is PKCβ, a component of the VEGF signaling pathway, which promotes tumor angiogenesis. PKCβ over-expression and elevated activity is observed in a variety of cancer types [1]. The serine/threonine kinase GSK3β, a target of PKCβ, is a key regulator of multiple signaling pathways [2] and is also often activated in tumors. Phosphorylation of GSK3β by PKCβ promotes cell proliferation by inhibiting cell cycle regulators [3]. In addition, PKCβ suppresses apoptosis by phosphorylating GSK3β on Serine 9 and promotes (endothelial) cell proliferation [4,5]. The PKC signaling pathway plays an important role in tumor-induced angiogenesis, tumor growth, differentiation, cytokine secretion, migration and apoptosis.

Enzastaurin (LY317615.HCl) is an ATP-competitive selective inhibitor for PKCβ [6,7]. It is known, that Enzastaurin reduces cell proliferation by inhibition of the PKCβ signaling pathway [7]. The activity of the serine/threonine kinase AKT, which promotes cell proliferation by inhibiting cell cycle inhibitors [3], is reduced under these circumstances via the inhibition of PKCβ [8]. Enzastaurin is used as a single agent or in combination with chemotherapeutic agents, targeted therapies or irradiation (combination with Pemetrexed [9], with Gemcitabine or Cisplatin [10] or with irradiation [4,11,12]). There are also successful trials in combination with other targeted GSK3β inhibitors [13] or with anti-neoplastic agents [14]. Enzastaurin is currently investigated in clinical trials phase I, II and III, because it is well-tolerated and first results suggested it to serve as a good platform for combinational drug therapies [15-17].

The second target gene, which attracts increasing attention in the fields of signaling research and cancer therapy, is the serine/threonine kinase Plk1 (polo-like kinase 1) [18], because it shows elevated activity in all human tumors [19-21]. Plk1 plays a pivotal role for mitosis especially of cancer cells and thus as a measure for the aggressiveness of a tumor [22]. Plk1 has predictive and prognostic value for patients with diverse cancers [22,23]. The importance of Plk1 as a measure for the aggressiveness of a tumor results from its important role for the mitotic checkpoints of cancer cells [24-28]. Interfering with Plk1 activity and/or expression with dominant-negative mutants, antibody microinjection, antisense oligonucleotides or small interfering RNAs leads to different mistakes in centrosomal maturation, mitotic catastrophe, increased apoptosis and tumor inhibition in cancer cells [23,27,29-38]. In addition to its role during mitosis, Plk1 has multiple functions outside of mitosis. Plk1 is for example necessary for checkpoint recovery after DNA damage [23,39] and is required for G1/S phase [40].

SBE13 is a selective type II Plk1 inhibitor which is able to induce a delay in cell cycle progression, to reduce cell proliferation and to induce apoptosis in a broad range of human cancer cell lines [33,34]. SBE13 displayed 1,000-fold selectivity towards Plk family members with only marginal reduced kinase activity of Plk2 and Plk3 and did not influence Aurora A activity. SBE13 displayed a differential effect between cancer and primary cells [41], confirming earlier studies using Plk1-specific siRNAs [35,38]. The PKCβ inhibitor Enzastaurin also inhibits tumor cell proliferation, but not of untransformed colonic epithelial cells [42]. For that reason, the combination of Plk1 and PKCβ inhibitors might be a promising tool in cancer therapy.

The aim of the current study was to investigate the effects of the combination of Enzastaurin with the Plk1 inhibitor SBE13 on the induction of apoptosis, the reduction of cancer cell proliferation, and on the cell cycle distribution of cancer cells and one immortalized, but not transformed cell line. Furthermore we analyzed the effects of the combined PKCβ and Plk1 inhibition on the protein expression of important target proteins. We used different cancer cell lines including the isogenic HCT116^p53wt^ and HCT116^p53-/-^ cells and hTERT-RPE1 cells, because they have a different p53 status.

## RESULTS

### PKCβ expression levels in HeLa and MCF-7 cells

PKCβ over-expression and elevated activity is observed in a variety of human tumors. In first studies, we did western blot analyses with untreated control lysates to elucidate differences in PKCβ expression in HeLa and MCF-7 cells, demonstrating higher PKCβ protein expression in HeLa cells compared to MCF-7 cells (data not shown). Next we investigated the effect of Enzastaurin on PKCβ protein levels in HeLa cells to determine whether the PKCβ inhibitor influences not only the activity, but also the protein levels of PKCβ. As expected, total PKCβ protein was not affected by the treatment with either Enzastaurin alone or with the combination of Enzastaurin and SBE13 (data not shown).

### Reduced phosphorylation of GSK3β on S9 in HeLa and MCF-7 cells

**Figure 1:**
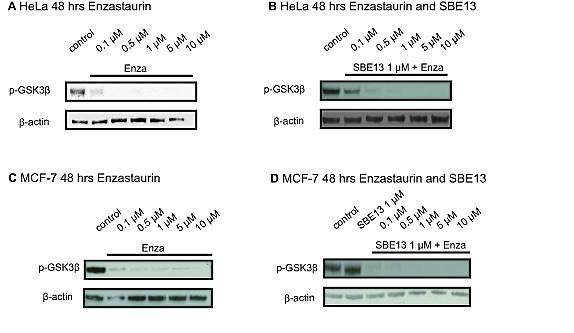
Western Blot analyses of pGSK3β protein levels in HeLa and MCF-7 cells after treatment with Enzastaurin and SBE13 Western Blot analysis of pGSK3β protein levels in HeLa cells 48 hrs (A) after treatment with Enzastaurin and after treatment with Enzastaurin in combination with 1 μM SBE13 (B), and pGSK3β protein levels in MCF-7 cells 48 hrs after treatment with Enzastaurin (C) and 48 hrs after treatment with Enzastaurin in combination with 1 μM SBE13 (D). Figures show representative blots

In untreated cells GSK3β is a target of PKCβ and it is phosphorylated on serine 9 by PKCβ to prevent apoptosis. To analyze whether Enzastaurin inhibits PKCβ activity in HeLa and in MCF-7 cells, we determined phospho-GSK3β protein after treatment with increasing Enzastaurin concentrations and with the combination of Enzastaurin with SBE13. We observed a reduction of GSK3β phosphorylation in both cell lines (Figure 1). Treatment of HeLa cells with Enzastaurin reduced the phosphorylation of GSK3β 48 and 72 hours after treatment (Figure 1A and data not shown). The combinatorial treatment of HeLa cells with Enzastaurin and SBE13 had no additional effect on the inhibition of the GSK3β phosphorylation 48 and 72 hours after treatment (Figure 1B and data not shown) compared to the treatment with Enzastaurin alone. Treatment of MCF-7 cells with Enzastaurin for 24, 48 and 72 hours resulted also in a reduced phosphorylation of GSK3β in a time and dose-dependent manner (Figure 1C and data not shown). As observed in HeLa cells the combinatorial treatment with the two inhibitors Enzastaurin and SBE13 did not improve the reduced GSK3β phosphorylation (Figure 1D and data not shown).

To confirm that the reduced levels of phospho-GSK3β in the Western blot analyses were due to reduced phosphorylation and not based on reduced total GSK3β protein, we determined the levels of GSK3β protein in both cell lines after treatment with Enzastaurin. The expression of non-phosphorylated GSK3β was not influenced by the treatment with Enzastaurin 48 and 72 hours after treatment (data not shown).

### Effects of Enzastaurin alone and in combination with SBE13 on Plk1 expression in HeLa and MCF-7 cells

**Figure 2:**
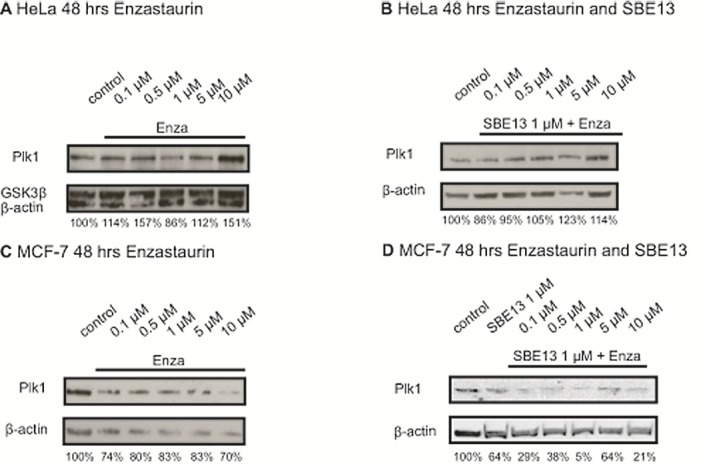
Western Blot analyses of Plk1 protein expression in HeLa and MCF-7 cells 48 hours after treatment with Enzastaurin and SBE13 Western Blot analysis of Plk1 protein expression in HeLa cells 48 hrs after treatment with Enzastaurin (A) and after treatment with Enzastaurin in combination with 1 μM SBE13 (B) and Plk1 protein expression in MCF-7 cells 48 hrs after treatment with Enzastaurin (C) and after treatment with Enzastaurin in combination with 1 μM SBE13 (D). Figures show representative blots and the numbers represent relative Plk1 protein levels referring to untreated controls as 100%.

We did western blot analyses to determine the Plk1 expression in both cell lines after treatment with Enzastaurin and SBE13 (Figure 2).

In HeLa cells we observed an increase in Plk1 protein levels up to 151% with 10 μM Enzastaurin compared to untreated control cells after 48 hours (Figure 2A). The combinatorial treatment did not influence the Plk1 protein levels (Figure 2B) 48 hours after treatment compared to untreated control cells and did not elevate Plk1 protein compared to single Enzastaurin treatment.

In MCF-7 cells in contrast there was a strong decrease in Plk1 protein levels detectable (Figure 2C, D). Cells treated with increasing concentrations of Enzastaurin showed reduced Plk1 expression levels down to 70% with 10 μM Enzastaurin compared to the control cells (Figure 2C). The combinatorial treatment with Enzastaurin and SBE13 reduces Plk1 protein levels compared to cells treated with Enzastaurin alone very strongly to 21% with 10 μM Enzastaurin in combination with 1 μM SBE13, which corresponds to the observed G0/G1 arrest (see below, Figure 2D).

### Cell Cycle Analysis of HeLa, MCF-7, hTERT-RPE1 and HCT116p^53wt^ and HCT116^p53-/-^ cells after treatment with Enzastaurin and SBE13

We did FACScan analyses to determine the cell cycle distribution of HeLa, MCF-7, hTERT-RPE1, and HCT116^p53wt^ and HCT116^p53-/-^ cells to examine whether the changes in Plk1 expression were associated with an arrest in particular stages of the cell cycle and to analyze the influence of PKCβ inhibition on cell cycle distribution. In addition, we wanted to figure out whether the absence or presence of functional p53 influences the cell cycle arrest.

**Figure 3:**
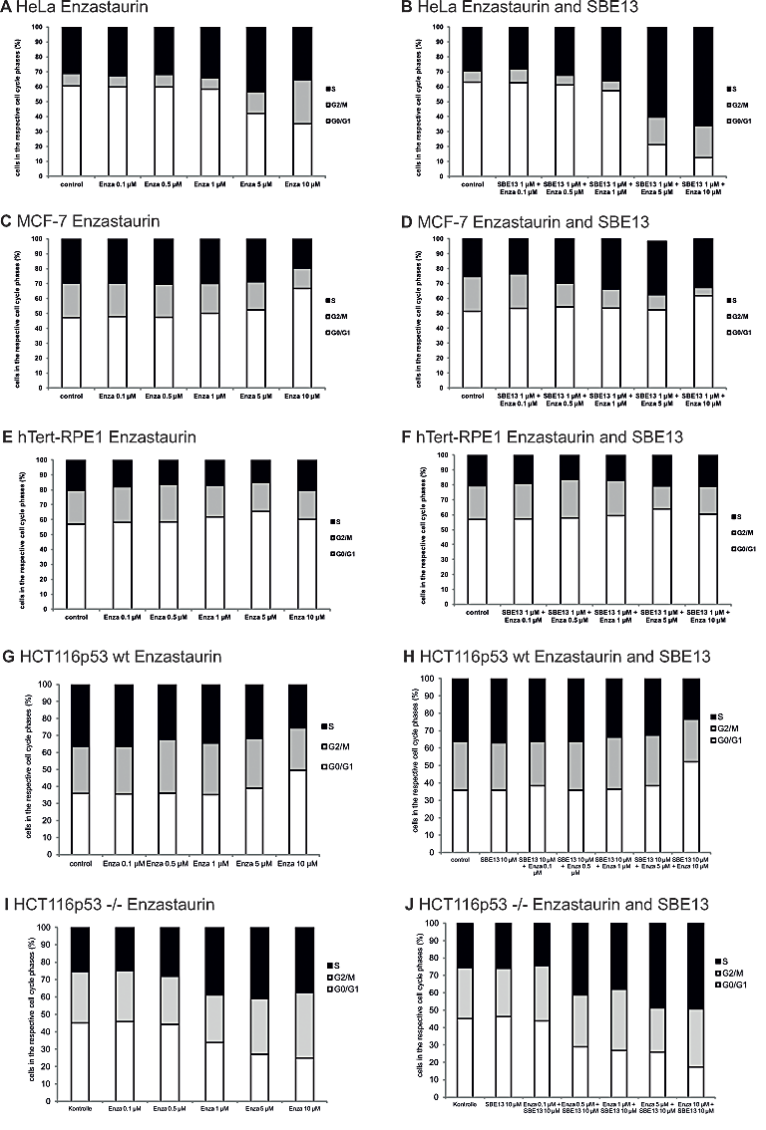
Effect of Enzastaurin and SBE13 on cell cycle distribution of HeLa, MCF-7, hTERT-RPE1 and HCT116p^53wt^ and HCT116p^53-/-^ cells Cells were incubated for 48 or 72 hours with Enzastaurin alone (A, C, E, G, I) or in combination with SBE13 (B, D, F, H, J) and analyzed for their cell cycle distribution. The graphs show the relative number of cells in the respective cell cycle phases.

First, we analyzed HeLa and MCF-7 cells and observed crucial differences in their cell cycle distribution 72 hours after treatment with the two inhibitors (Figure 3A-D). We observed a G2/M arrest in HeLa cells: after 72 hours 14% of the cells treated with 5 μM Enzastaurin were in G2/M-Phase and treatment with 10 μM Enzastaurin increases the number of cells in G2/M phase up to 30% compared to 8% of control cells in the G2/M phase (Figure 3A). In addition to the observed G2/M arrest, treatment with 5 μM Enzastaurin also enhances the amount of cells in S phase up to 43% (control cells: 31%). Cells treated with the combination of Enzastaurin and SBE13 (Figure 3B) displayed a stronger arrest in S phase compared to cells treated with Enzastaurin alone up to 60% with 5 μM Enza + 1 μM SBE13 and up to 66% with 10 μM Enza + 1 μM SBE13. The combination of 10 μM Enzastaurin with 1 μM SBE13 enhances the number of cells in G2/M phase compared to control cells, but reduces the number of cells in G2/M phase compared with the treatment with 10 μM Enzastaurin alone (control cells 7% in G2/M, 10 μM Enza + 1 μM SBE13 21% cells in G2/M).

In MCF-7 cells we did not observe a G2/M arrest after treatment with Enzastaurin and SBE13 in all analyzed concentrations and combinations, instead we could detect a G0/G1 enrichment 72 hours after treatment with 10 μM Enzastaurin alone and in combination with SBE13 (Figures 3C, D). Treatment with 10 μM Enzastaurin led to an increase of cells in G0/G1 phase up to 66% (control cells: 47% G0/G1) (Figure 3C). The combination of 10 μM Enzastaurin with 1 μM SBE13 enhances the amount of cells in G0/G1 phase up to 61% (control cells. 51%) (Figure 3D). This G0/G1 arrest was accompanied by a dose-dependent decrease of cells in the G2/M phase, the treatment with 10 μM Enzastaurin in combination with 1 μM SBE13 leads to the strongest decrease of cells down to 5% in the G2/M phase compared to 23% control cells in the G2/M phase and an increase of cells in S phase (control cells: 25%, 10 μM Enzastaurin together with 1 μM SBE13: 34%).

Next we analyzed the immortalized, but not transformed hTERT-RPE1 cells, to compare effects in cancer cells with non-cancer cells. The cell cycle distribution of hTERT-RPE1 cells was not altered after the treatment with 1 μM SBE13, increasing concentrations of Enzastaurin or the combination of both inhibitors (Figures 3E and 3F).

To further investigate the dependence of cell cycle arrest on the p53 status of the cells, we did cell cycle analyses in HCT116^p53wt^ and HCT116^p53-/-^ cells after treatment with the inhibitors as single agents or in combination, respectively. These cells nicely confirmed the initial observation in MCF-7 vs. HeLa cells, showing an increasing amount of cells in G0/G1 phase in HCT116^p53wt^ cells (Figures 3 G, H), and an increasing amount of cells in S and G2/M phase in HCT116^p53-/-^ cells (Figures 3 I, J).

### Analysis of apoptosis induction in HeLa and MCF-7 cells after treatment with Enzastaurin and SBE13

**Figure 4:**
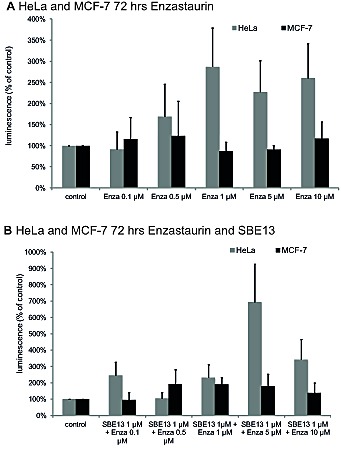
Caspase 3/7 assays in HeLa and MCF-7 cells 72 hrs after treatment with Enzastaurin and SBE13 Graphical summary of Caspase 3/7 activation in HeLa and MCF-7 72 hours after treatment with Enzastaurin alone (A) or in combination with SBE13 (B). Luminescence is given as RLU (relative light unit) referring to untreated controls as 100% (n=3, mean ± SD).

We did caspase 3/7 assays, because a G2/M arrest is often followed by apoptosis [43] and PKCβ is also involved in apoptotic pathways [44].To analyze whether the combination of Enzastaurin and SBE13 enhances the induction of apoptosis, we did caspase 3/7 assays (Figure 4). The combination of the two inhibitors enhances the induction of apoptosis in HeLa cells compared to Enzastaurin alone (5 μM Enzastaurin: 227%, 10 μM Enzastaurin: 260%, 5 μM Enzastaurin + 1 μM SBE13: 693%, 10 μM Enzastaurin + 1 μM SBE13: 342%). In MCF-7 cells the increase in apoptosis induction was much weaker than in HeLa cells. However the combination of Enzastaurin with SBE13 led to a slightly elevated activity of caspases 3/7 compared to the treatment with Enzastaurin alone (10 μM Enzastaurin: 116%, 10 μM Enzastaurin + 1 μM SBE13: 138%)

### Cell proliferation analysis of HeLa, MCF-7, hTERT-RPE1 and HCT116p^53wt^ and HCT116p^53-/-^ cells after treatment with Enzastaurin and SBE13

**Figure 5:**
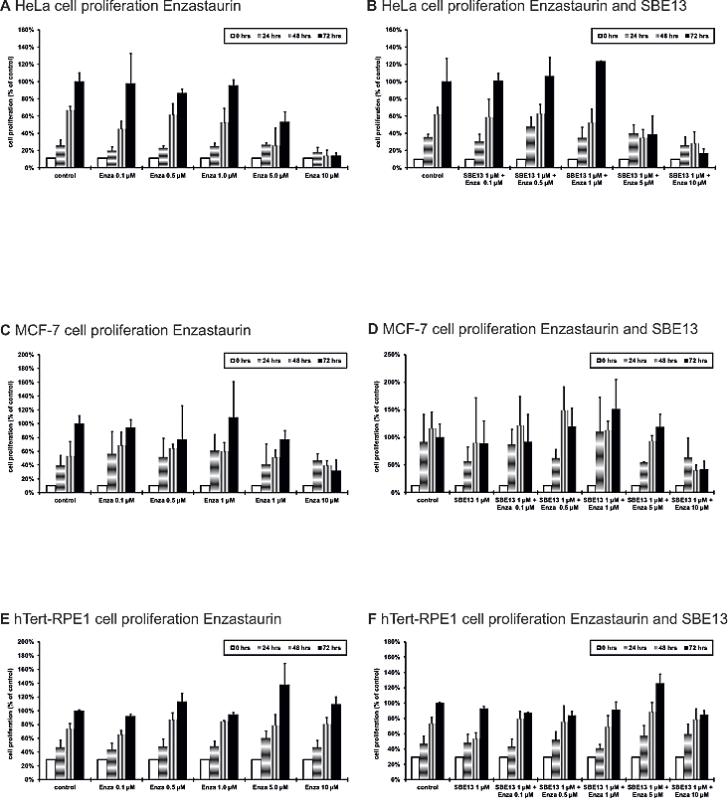
Cell proliferation of HeLa, MCF-7 and hTERT-RPE1 cells 24-72 hours after treatment with Enzastaurin and SBE13 Cells were incubated for 24-72 hours with Enzastaurin alone (HeLa cells: A; MCF-7 cells: C, hTERT-RPE1 cells: E) or in combination with SBE13 (HeLa cells: B; MCF-7 cells: D, hTERT-RPE1 cells: F). Percentage of surviving cells is given as percentage of the number of control cells after 72 hrs. Bar graphs represent means of three different experiments.

To investigate whether the cell cycle arrest and induction of apoptosis causes a reduction of cell proliferation, we analyzed the cell proliferation of the various cell lines after treatment with Enzastaurin alone and in combination with SBE13 (Figure 5).HeLa cells showed a significant reduced cell proliferation to levels of 53% with 5 μM Enzastaurin (p=0.047) and 14% with 10 μM Enzastaurin (p=0.0036) (Figure 5A). In combination with SBE13 we observed a synergistic reduction to 39% with 5 μM Enzastaurin + 1 μM SBE13 and to 17% with 10 μM Enzastaurin + 1 μM SBE13 (p=0.025, CI=0.83, Figure 5B).MCF-7 cells displayed a moderate reduction of proliferation compared to the reduction in HeLa cells. MCF-7 cells showed a reduction in cell proliferation to 77% with 5 μM Enzastaurin and to 32% with 10 μM Enzastaurin (p=0.033) (Figure 5C). The combination with SBE13 showed no additional effect on cell proliferation (Figure 5D).

To determine, whether this effect was cancer cell-specific we analyzed the immortalized, but not transformed hTERT-RPE1 cells. The cell proliferation of hTERT-RPE1 cells was not decreased after the treatment with SBE13, Enzastaurin or the combination of both inhibitors (Figures 5E and 5F).

**Figure 6:**
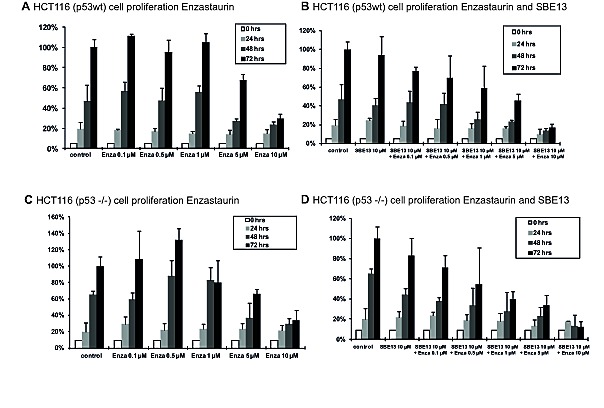
Cell proliferation of HCT116p^53wt^ and HCT116p^53-/-^ cells 24-72 hours after treatment with Enzastaurin and SBE13 Cells were incubated for 24-72 hours with Enzastaurin alone (A and C) or in combination with SBE13 (B and D). Percentage of surviving cells is given as percentage of the number of control cells after 72 hrs. Bar graphs represent means of three different experiments.

To investigate whether the observed effects are due to the p53 status of the cells, we also examined HCT116^p53wt^ and HCT116^p53-/-^ (Figure 6). Enzastaurin alone reduces the cell proliferation of HCT116^p53wt^ cells with an *EC_50_* of 7.2 μM, the combination with SBE13 lowers this *EC_50_* to 4 μM (Figures 6A and 6B). This enhanced reduction of cell proliferation was synergistic (CI=0.82). The *EC_50_* value of Enzastaurin in HCT116^p53-/-^ cells was comparable (7.4 μM), the combination reduces the *EC_50_* value much stronger than in the HCT116^p53wt^ cells (0.6 μM, CI=0.21, Figures 6C and 6D).

These results confirm the hypothesis that the enhanced reduction in cell proliferation after treatment with SBE13 and Enzastaurin is due to missing p53 function of the cells, because in contrast to the former comparison of HeLa and MCF-7 cells the HCT116 cells only differ in their p53 status.

## DISCUSSION

In the current study we analyzed for the first time the effects of PKCβ inhibition using Enzastaurin in combination with Plk1 inhibition using SBE13 on cell cycle regulation and induction of apoptosis in different cancer cell lines and in immortalized, but not transformed hTERT-RPE1 cells.

For the first studies, we used HeLa and MCF-7 cells because they have different p53 status and showed also differences in their PKCβ expression. In all analyses, MCF-7 cells were less sensitive than HeLa cells to the inhibitor treatments, suggesting the importance of an intact p53 function. To analyze the influence of the two inhibitors on cell cycle regulators, we did western blot analyses. Treatment with Enzastaurin or SBE13 did not influence the PKCβ or GSK3β expression in HeLa cells. The phosphorylation of GSK3β on S9 by PKCβ could be inhibited by treatment with Enzastaurin both in HeLa and MCF-7. This is in concordance with the literature, because Enzastaurin inhibits the PKCβ activity and thereby the phosphorylation of GSK3β on S9 [5].

The Plk1 protein level in HeLa cells was elevated after treatment with Enzastaurin alone and in combination with SBE13. This could be an indirect consequence of the observed G2/M arrest, because the Plk1 expression peaks at G2/M phase, or a direct effect on the cell cycle regulation. In MCF-7 cells we could not observe an increase in Plk1 protein levels, instead the Plk1 protein level decreases. Thus, the observed changes of Plk1 protein levels after treatment with Enzastaurin and SBE13 alone and in combination are in concordance with our FACScan analyses: MCF-7 cells do not arrest in G2/M phase, but in G0/G1 phase.

So the different Plk1 expression levels directly reflect the different cell cycle arrest of HeLa vs. MCF-7 cells giving a first hint that this might be p53-dependent. This observation is in concordance with earlier studies from other groups correlating the reaction of cancer and primary cells after treatment with microtubule poisons to their p53 status, where p53 wild-type cells were resistant to the chemotherapy, but p53-deficient cells were sensitive to the treatment [45-49]. In our study, the p53-deficient HeLa and HCT116^p53-/-^ cells for example showed a G2/M arrest after Enzastaurin treatment alone and an additional increase of cells in S-Phase after combination with SBE13. A possible explanation could be that the p53-deficient cells are not able to repair their DNA damage induced by the Plk1 inhibition at the G1/S checkpoint because of their loss of intact p53 function, so they are forced to begin mitosis with unrepaired DNA damage, resulting in an elevated number of cells in S and in G2/M phase. Cells with intact p53 function (MCF-7 and HCT116^p53wt^) showed an increased number of cells in G0/G1 phase, obviously arresting at the G1/S transition. These observations are in concordance with other studies, linking the reaction of cells after DNA damage to their p53 status [50]. The first study which showed how the p53 status affects the effects of Plk1 inhibition revealed that normal non-transformed MCF10A and hTERT-RPE1 cells tolerate depletion of Plk1 pretty well compared to different cancer cell lines, and that co-depletion of p53 in MCF10A cells rendered them to be extremely sensitive to Plk1 inhibition [51]. Later, it was analyzed by the same group how Plk1 regulates p53. The regulation of p53 by Plk1 was further established by the identification of two p53 regulators, Topors and GTSE1, as direct Plk1 substrates [52]. Plk1 phosphorylates Topors promoting its E3 ubiquitination activity towards p53, but inhibits its sumoylation activity towards p53, thus contributing to p53 degradation. Moreover, Plk1 phosphorylates GTSE1 which then translocates p53 from the nucleus to the cytoplasm. This leads to the exposure of p53 to the proteasome degradation machinery [53]. Another approach could correlate epigenetic modulations of the Plk family under oxidative stress to the p53 status of cancer cells, indicating the importance of analyzing p53 before potential Plk1-specific therapies [54].

In addition to the cancer cells, we analyzed the immortalized, but not transformed hTERT-RPE1 cells to investigate, whether we observe a cancer cell-specific effect and to analyze another p53 wild-type cell line. The cell cycle distribution of hTERT-RPE1 cells was not influenced by the treatment with 1 μM SBE13, increasing concentrations of Enzastaurin or the combination of both inhibitors. This is in concordance with our own studies, because it is already known that 1 μM SBE13 does not alter the cell cycle distribution of hTERT-RPE1 cells [41]. Enzastaurin is also a highly selective inhibitor, which does not impair the cell proliferation of non-transformed colonic epithelial cells [42].

The different types of cell cycle arrest could be observed in other studies dealing with NEDD inhibitors [55]. They observed that HCT116^p53wt^ cells with functional p53 arrested at the G1/S transition after NEDD inhibition and the HCT116^p53-/-^ cells without a functional p53-dependent G1/S checkpoint arrested later in mitosis due to the formation of monopolar spindles. Using BI2536 [56,57] the authors observed comparable results as with NEDD inhibition.

Because a G2/M arrest is often followed by apoptosis [43], we analyzed whether the G2/M arrest in HeLa cells induced by the combinatorial treatment with Enzastaurin and SBE13 is also followed by apoptosis and how the p53-proficient MCF-7 cells acted, because they showed no enrichment of cells in the G2/M phase. As expected, HeLa cells showed strong induction of apoptosis, but MCF-7 cells with their functional G1/S checkpoint did not undergo apoptosis, because they are able to repair the DNA damage induced by Plk1 inhibition and complete mitosis normally. These observations again are in concordance with Tillement et al. [55]. In both cell lines we observed an elevated induction of apoptosis with the combination of the two inhibitors compared to Enzastaurin treatment alone. Earlier studies could show that Plk1 inhibition by siRNAs or ASOs elevates drug sensitivity of cancer cells [35,58]. Thus we could confirm the chemo-sensitizing effect of Plk1 inhibition using SBE13, which sensitizes the cells to the Enzastaurin treatment.

Enzastaurin and SBE13 are known to reduce cell proliferation in different cancer cell lines. Thus, to investigate whether the observed cell cycle arrest and the induction of apoptosis caused a reduction of cell proliferation we analyzed the effect of the combinatorial treatment with Enzastaurin and SBE13 on cell proliferation of HeLa, MCF-7 and hTERT-RPE1 cells and HCT116^p53wt^ and HCT116^p53-/-^ cells. In HeLa cells SBE13 was able to enhance the effect of Enzastaurin in reducing cell proliferation synergistically. In our earlier studies, we could already show that inhibition of Plk1 sensitizes cancer cells to anti-neoplastic drugs [35,58]. Now we were able to show this sensitizing effect using a small molecule inhibitor, SBE13, together with Enzastaurin. In MCF-7 cells in contrast, there was no additional effect of SBE13 treatment on cell proliferation reduction compared to Enzastaurin alone. In addition, the reduction of cell proliferation was much less pronounced than in HeLa cells, which could be due to their functional p53 whereas HeLa cells are forced to go further in the cell cycle and to start mitosis with damaged DNA and arrest in G2/M phase followed by apoptosis. As observed in the cell cycle analyses hTERT-RPE1 cells are not influenced by the treatment of SBE13 together with Enzastaurin, the cell proliferation remains unchanged. The finding, that the combination of a Plk1 and a PKCβ inhibition reduces the cell proliferation of cancer but not of primary cells could be of great importance for the development of future anti-cancer therapy strategies.

We also did experiments using HCT116^p53wt^ and HCT116^p53-/-^ cells to ensure that the differences observed after treatment of HeLa and MCF-7 cells with the combination of Enzastaurin and SBE13 are due to the different p53 status of the cells. The HCT116^p53-/-^ cells showed a stronger reduction of the cell proliferation after the treatment with the two inhibitors than the HCT116^p53wt^ cells.

Apparently, a functional p53 protects cells from the cytotoxic effects caused by the combinatorial treatment with Enzastaurin and SBE13. Thus, this combination can be very useful to treat p53-deficient cancers, while it displays no toxicity to normal cells due to their functional p53. A very interesting approach regarding the susceptibility of cancers to chemotherapeutics dependent on their p53 status has been developed in earlier studies by Blagosklonny et al. They describe the possibility to pretreat cells with DNA-damaging agents before adding microtubule drugs leading to selective killing of cells with defective p53/p21-dependent checkpoint [45,46]. In general, the induction of wild-type p53 protects normal cells in culture from cytotoxicity caused by conventional cancer therapeutics, especially together with S- or M-phase poisons. This so called p53-dependent cyclotherapy using p53 activators stops proliferation of normal cells / healthy tissues via cell cycle arrest, while leaving the p53-deficient tumor susceptible to the conventional chemotherapy (DNA-damaging agents, microtubule poisons) [47-49].

Taken together, our experiments nicely confirmed our hypothesis that the p53 status of cancer cells could serve as a predictive marker, which can be used to select patients who will profit from a combinatorial Plk1 and PKCβ inhibition therapy, especially to treat the p53-deficient cancers.

## METHODS

### Kinase inhibitors and antibodies

The Plk1 kinase inhibitor SBE13 was purchased from the SPECS compound catalogue (Delft, Netherlands), PKCβ kinase inhibitor Enzastaurin (LY317615.HCl) was purchased from Selleck (Absource Diagnostics GmbH München, Germany).

Monoclonal-anti-PKCβ, anti-GSK3β, monoclonal anti-Plk1 antibodies, goat anti-mouse and goat anti-rabbit secondary antibodies were from Santa Cruz Biotechnology, Inc., (Heidelberg, Germany), anti-phospho-GSK3β antibody was from Cell Signaling (Frankfurt/Main, Germany) and monoclonal β-actin-antibody from Sigma-Aldrich (Taufkirchen, Germany).

### Cell culture

The cancer cell lines HeLa and MCF-7 were from DSMZ (Braunschweig, Germany), hTERT-RPE1 cells were from Clontech (Saint-Germain-en Laye, France). All cells were cultured according to the supplier's instructions without antibiotics. Fetal calf serum (FCS) was from PAA Laboratories (Cölbe, Germany), DMEM, RPMI 1640, phosphate buffered saline (PBS), glutamine, and trypsin were from Invitrogen (Karlsruhe, Germany). HCT116^p53+/+^ and HCT116^p53^ cells were cultured as instructed.

### Treatment and analysis of cancer cells

Cells were treated with SBE13 and Enzastaurin alone or in combination one day after subculturing. Cells were seeded onto 6-well-plates, or 75-cm^2^- flasks, respectively. Control cells were incubated with normal culture medium without antibiotics. Concentrations of SBE13 ranged from 1 μM–10 μM, Enzastaurin concentrations ranged from 0.1 μM–10 μM. The growth rate of 1x10^5^ cells per 6-well was determined by counting cells at 24 to 72 hours after treatment. Cell culture studies were performed in triplicate for each time point. Cells were harvested 0-72 hours after treatment for further analyses.

### Western blot analysis

Total protein (50 μg) was separated on 10% Bis-Tris-polyacrylamide gels and transferred (at 30 V for 1 hr) to Immobilon™-P membranes (Millipore, Bedford, MA) according to the Invitrogen protocol (Karlsruhe, Germany). Membranes were incubated for 1 hr in 5% powdered nonfat milk in PBS with antibodies against PKCβ (1:200 - 1:1,000), GSK3β (1:2,500), p-GSK3β (1:1000), Plk1 (1:200), or β-actin (1:100,000) and for 30 min in 5% nonfat dry milk with goat anti-mouse or goat anti-rabbit serum (1:2,000) and visualized as described [38].

All protein expression levels were presented as described [38], scanned and quantified with the freeware ImageJ (National Institutes of Health, USA).

### FACS analysis

Cell cycle distribution was analyzed using a FACScalibur apparatus (Becton Dickinson, Heidelberg, Germany). Quantification was carried out using ModFit LT 3.2 for MAC (Verity Software House, Topsham, ME). For FACS analysis, cells were harvested at the indicated time points, washed with PBS, fixed and stained as described [58]. For each experiment, 30,000 cells were analyzed in triplicate.

### Caspase Assay

We did Caspase-Glo^®^ assays to detect activation of Caspase 3/7 using the Caspase-Glo^®^ 3/7 Assay System (Promega, Mannheim, Germany). In brief, cells were analyzed 24-72 hrs after treatment with Enzastaurin and SBE13. 10 μg total protein were mixed with the Caspase-Glo^®^ substrate, incubated for 30 min and analyzed using a Victor™ 1420 multilabel counter (Perkin Elmer Wallac, Freiburg, Germany). The emitted light is measured at 562 nm referred to as RLU (relative luminescence units).

### Statistical methods

All experiments were performed at least in triplicate. All treatments were compared with untreated control cells. Statistical analysis was performed with student's t-test to consider random effects as described [38]. *EC_50_* values were calculated from the cell proliferation experiments assuming the cell number of control cells at the latest time point as 100%.

The combination index was calculated using the following equation: c.i. = (Am)_50_/(As)_50_ + (Bm)_50_/(Bs)_50_, where (Am)_50_ is the concentration of drug A necessary to achieve a 50% inhibitory effect (*IC_50_*) in the combination, (As)_50_ is the concentration of the same drug that will produce the identical level of effect alone, (Bm)_50_ is the *IC_50_* of drug B in the combination and (Bs)_50_ is the *IC_50_* of drug B after single administration. Antagonism is indicated when c.i.>1, c.i.=1 indicates an additive effect and a c.i.<1 indicates synergy [59].
